# How Do Viral and Host Factors Modulate the Sexual Transmission of HIV? Can Transmission Be Blocked?

**DOI:** 10.1371/journal.pmed.0030079

**Published:** 2006-02-28

**Authors:** Kalpana Gupta, Per Johan Klasse

## Abstract

A better understanding of sexual transmission, say the authors, will enable more rational design of vaccines and microbicides and potential combinations of the two.

In 2004, about 3 million people died of AIDS [ 1]. Because sexual intercourse accounts for the vast majority of HIV transmissions worldwide, it is important to understand the events that occur in the genital or rectal mucosa during transmission. Here we dissect a number of factors in the transmitter and recipient that are relevant before, during, and after transmission. We weigh the evidence suggesting that the transmitted virus differs from the virus that predominates in the transmitter. We discuss the prospect for protection by innate and adaptive immune mechanisms at mucosal surfaces as well as by locally applied inhibitors of viral replication, *microbicides*. A better understanding of sexual transmission will enable more rational designs of vaccines and microbicides and potential combinations of the two.

## Transmitting Host, Infectiousness, and Transmitted Virus

Early studies estimated the rate of sexual HIV transmission to one infection per 1,000–2,000 coital acts. New data indicate that the rate varies widely with the phase of the infection, and is more than 10-fold higher during acute infection [ 2, 3]. Thus, interventions early in infection that reduce transmission might have the greatest impact on the epidemic.

It is still not clear whether HIV transmission is mediated mainly by cell-free virus or by infected cells. Both forms of virus are present in human semen and vaginal fluids [ 4]. What immune responses will protect may depend on whether mainly virus particles or infected cells mediate infection. Neutralization by antibodies is less efficient against cell- than virion-mediated infection [ 5]. Thus, results obtained with cell-free virus in animal vaccine and microbicide experiments may not allow extrapolation to protection against cell-associated virus [ 6].

Knowledge of the histological sources and properties of virus from semen and vaginal fluids is incomplete. The viral load in genital fluids is more variable, and in untreated individuals generally lower, than in blood. The compartmentalization [ 4] promotes local evolutionary viral divergence. In the genital tract of HIV-infected females, virus originates from the cervix and its associated lymphatic tissue, as well as from menstrual blood, but virus can also be recovered from the vagina of women who have had hysterectomies [ 4]. Semen of HIV-positive men contains both infected macrophages and lymphocytes [ 4]. In simian immunodeficiency virus (SIV)-infected male macaques, most of the virus in the genital tract is associated with the epididymides, but ejaculates from vasectomized men, and pre-ejaculatory fluid, can be infectious [ 4]; 10%–20% of men with undetectable blood viral load due to antiretroviral (ARV) therapy have detectable virus in the genital tract [ 4]. The explanation may be that HIV replicates in the genital tract, for example in macrophages that reside there. Despite this compartmentalization, viral strains used experimentally in vitro and in animal models have been isolated from sources such as brain, lung, or peripheral blood, usually many years after transmission. Such virus may differ from genital-tract virus in the transmitter and from the early outgrowth virus in the recipient.

In a Ugandan study of HIV-serodiscordant couples (i.e., one partner is HIV-infected and the other not), plasma viral load correlated with the risk of HIV transmission: ARV treatment that reduced the viral load in blood to less than 1,000 RNA copies per milliliter seemed to reduce transmission [ 2]. The relative contribution of blood-derived virus to the infectiousness of men and women would affect the preventive impact of ARVs. That impact may be defined in an ongoing trial [ 7] that compares the effectiveness of two ARV therapies at reducing sexual transmission in HIV-serodiscordant couples.

Other sexually transmitted diseases (STDs) increase the amount of virus in the genital fluids of the transmitter, thereby raising transmission rates, particularly from men to women. STDs can increase the number of infected lymphocytes in the mucosa and enhance virus expression through immune activation [ 3, 4].

Infectiousness of the transmitter is thus a composite result of viral load in genital fluids, the tropism of the virus (which can be ascertained in vitro), and the in vivo infectivity of that virus (which may be difficult to measure in vitro). Viral escape both from neutralization and cellular immunity may modulate the phenotype of the virus, including its transmissibility. Hence there may be several levels of selection, or bottlenecks, in the transmitter before virus reaches the recipient, expands, and can be detected ( Table 1).

**Table 1:**
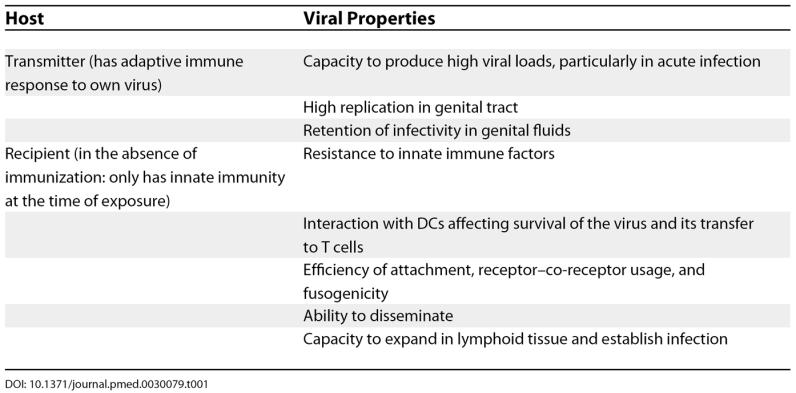
Potential Selection Pressures on Virus during Sexual Transmission

## Susceptibility of Host and Properties of Newly Transmitted Virus

**Figure 1:**
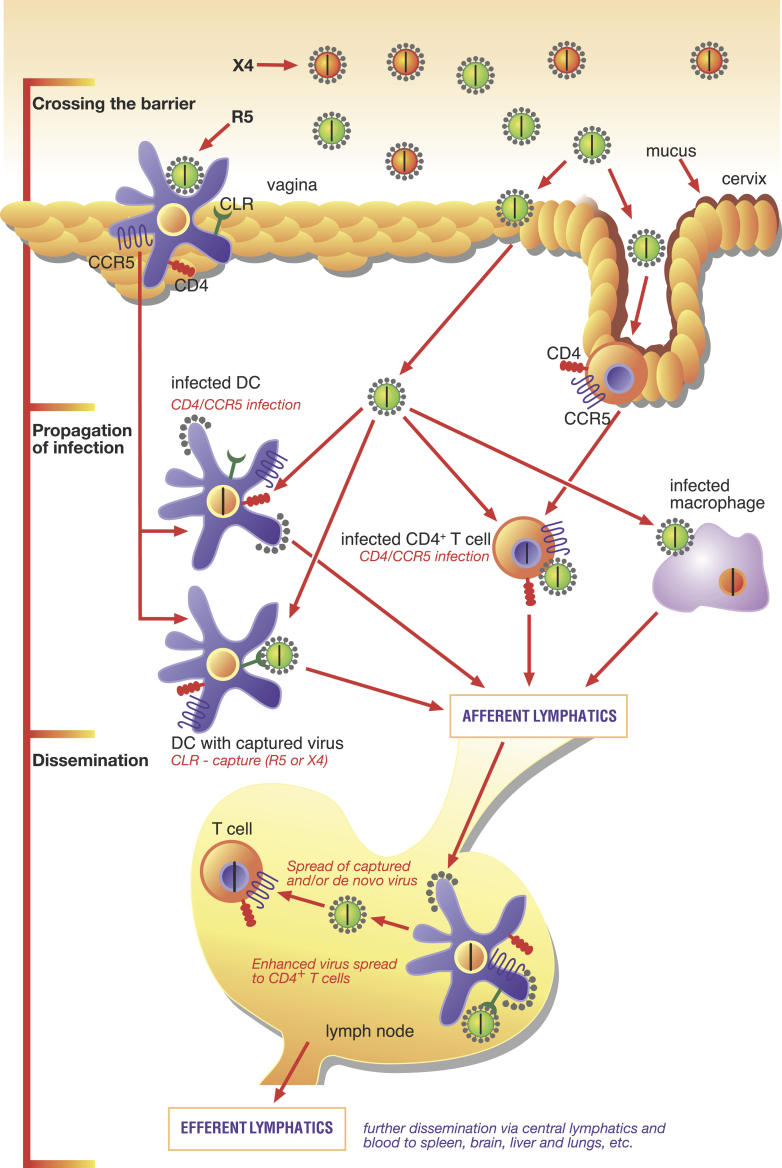
Early Events during Sexual Transmission of HIV From top to bottom: The epithelial lining of the vagina forms an efficient barrier to viral penetration when intact. Cervical mucus may serve to strengthen this barrier by physically trapping virions [ 25]. HIV crosses the epithelial barrier either because of epithelial damage (e.g., microabrasions and traumatic breaches or lesions caused by STDs), or capture by intra-epithelial DCs that convey the virus to target cells deeper in the mucosa [ 9]. In the lumen of the vagina, continuous with the cervical canal, virions with two kinds of tropism are illustrated: (i) X4 virus (orange) uses the co-receptor CXCR4 (rarely found early after transmission) and (ii) R5 virus (green) utilizing the co-receptor CCR5 (preferentially found early after transmission). Why R5 virus comes to dominate in the newly infected host is not known; it may reflect preferential amplification at a stage after transmission (not shown) [ 26]. To the left, an R5 virion is shown bound to an embedded DC, which has CD4, CCR5, and C-type lectin receptors on its surface, all of which can interact with the surface glycoprotein of the virus. The DC may merely capture X4 or R5 virus and carry it across the epithelial barrier or get infected by R5 virus and produce progeny virus (virus budding from the cell surface is shown as half-circular sections studded with grey Env spikes) [ 9]. To the right, an R5 virion binds to and infects a T helper lymphocyte, which has both CD4 and CCR5 on its surface. Virus that has penetrated into the epithelium is also shown to infect a macrophage. Arrows indicate how virus infects the first target cells and how progeny virus or DCs then migrate via the afferent lymphatics to reach the lymph nodes. Here, further amplification occurs in an environment rich in CD4 ^+^ target cells. From there, new generations of progeny virus cascade to the next level of lymphatic tissue. The gut-associated lymphoid tissue provides an important reservoir of susceptible cells that the virus rapidly decimates (not shown). Ultimately, the virus disseminates via efferent lymphatics and blood to spleen, brain, liver, and lungs. (Illustration: Courtesy *IAVI Report*, Volume 8, May–August 2004).

After deposition of the virus on the recipient mucosa, the outcome is affected by properties of the virus: its retention of infectivity while traversing the mucus and extracellular matrix, its affinity for entry receptors, the fusogenicity of its Env protein, and the efficiency of its interaction with dendritic cells (DCs) that can promote virus spread to, and amplification in, CD4 ^+^ T cells, locally and possibly at distant sites ( Figure 1) [ 8, 9]. Superimposed upon this, there are likely to be strong selective pressures in the recipient at later steps, determining which virus takes over in the newly infected host ( Table 1).

STDs make recipients more susceptible by breaking down the mucosal barrier in the case of genital ulcer disease and by increasing the number of susceptible cells in the mucosa through inflammation. Bacterial vaginosis—the colonization of the vagina by anaerobic bacteria—may further enhance the infectivity of incoming virus by raising the pH of the vaginal fluid: a higher pH would lead to slower virus inactivation and more efficient Env-mediated fusion [ 3, 4]. Male susceptibility can be reduced by two-thirds through adult circumcision [ 10]. It remains to be seen whether this is a direct effect on the number of target cells, in which the prepuce is rich, or an indirect effect on STD susceptibility and inflammation.

Newly transmitted viruses are likely to be more closely related to viruses that vaccines must protect against than later-stage viruses. Such early virus has been suggested to have distinct features. Early in infection, recipients appear to show much less viral diversity than the average infected person [ 11, 12]. Derdeyn et al. investigated eight newly infected people from a cohort of more than 1,000 continual exposures among discordant couples. These recipients harbored HIV strains (seven clade C and one clade G) with notably short V1 and V2 variable regions of the viral envelope glycoprotein gp120, which therefore had relatively few glycosylation sites. This could affect the recognition of the Env complex by neutralizing antibodies. Indeed, virus isolated from the recipient showed greater sensitivity to neutralization by sera from the transmitting individual than did virus from the transmitter [ 11]. The explanation may be either that neutralization-sensitive variants with more accessible receptor-binding domains pre-exist in the transmitter and are selectively transmitted, or that in the absence of neutralization pressure in the new host variants like this rapidly arise and expand.

However, such Env-selection does not seem to occur in clade-B transmission in intravenous-drug-abusing or homosexual cohorts [ 13]. It is uncertain whether this discrepancy would relate to modes of transmission, HIV subtypes, or viral properties that evolve as the proportion of acutely infected individuals changes in the course of the epidemic [ 13]. This is a topic of intensifying study that may shed new light on viral transmissibility.

Studies of highly exposed uninfected men from a cohort in Amsterdam failed to identify a unifying explanation for what prevented infection [ 14]. Different host factors may contribute to resistance to HIV-1 infection, including innate immunity. Lymphocytes from some of the men showed reduced R5 HIV infection in vitro due to autocrine secretion of CCL chemokines. Lymphocytes from other seronegative persons in the cohort also showed poor proliferative responses in vitro. A subset of individuals had low numbers of memory effector T cells and a low activation level of CD4 ^+^ T cells, thereby presenting few target cells for the virus [ 15]. The same magnitude and breadth of HIV-specific cytotoxic T cell responses were detected among exposed people whether they remained uninfected or later seroconverted [ 15]. Qualitative differences between the cellular immune responses may nevertheless render some protective and others merely indicative of exposure. This is another very active area of investigation that may define immune correlates of protection against HIV infection.

## Establishment of Infection in the Vagina or Rectum at a Histological Level

Experiments using cervical explants indicate that an intact mucosal epithelium forms an impenetrable barrier to HIV [ 8]. It is difficult to judge whether the barrier is equally impenetrable and the trafficking of cells across it similar when the tissue is perfused by circulating blood in the living organism. But there appear to be additional impediments to HIV replication, since replication is restricted even when the mucosal layer is breached by microabrasions.

In macaque models of SIV infection, the viral dose is higher than in most human exposures, perhaps higher even than when the transmitter is at the peak of primary-infection viral load. However, in this model the first infectious events in the mucosa occur within hours. During the first days, there is some dissemination beyond the mucosa: this may be mediated through lymphovascular anastomoses or trafficking of the virus by DCs [ 9]. It usually takes 4–6 days for the infection to become systemic. The establishment of a productive infection in the animal seems to depend critically on the continual seeding from a founder population of infected, ostensibly resting, memory CD4 ^+^ T cells in the genital tract. From these, the infection further disseminates to local lymph nodes and then to secondary lymphatic tissue, eventually becoming systemic [ 16, 17]. The escalation from one level to the next has been likened to a broadcast effect, each wave involving new bursts of amplification of the virus ( Figure 1) [ 16, 17]. Virus is found in the gut-associated lymphoid tissue by day 6–7, when only the rudiments of an SIV-specific immune response are detectable. Within 14 days of SIV infection, effector memory CD4 ^+^ cells in the intestinal lamina propria are selectively and permanently depleted [ 18]. It is plausible that many exposures to the virus that do not lead to established infections involve local, transitory viral replication. And when the infection does get established, scattered foci of founder cells arise from only a small fraction of the inoculum. The delay of up to six days before systemic replication, even in this high-dose model, and in spite of the rapid dissemination to draining lymph nodes, suggests that a crucial level of replication in local lymphoid tissues is required for establishment of infection. Below this level, the infection may abort. This scenario implies that prevention is still possible during a short period in which the virus is actively replicating. If this simian model accurately reflects the events in human transmission, it can help elucidate exactly what must be achieved in prevention.

Even though retroviral infection is seen as irreversible because of the integration of the pro-virus into the host-cell genome, the dynamic model of seeding and dissemination described above gives reason to modify that view: at the level of the organism, abortive HIV infections may be frequent. Sterilizing immunity would not need to achieve more than what hypothetically happens spontaneously in many cases when a seeding population is too small to sustain the cascading events. Consequently, there could be a window of opportunity early in infection for a potent, local, vaccine-induced immune response or a microbicide to prevent or dampen viral replication in the first host cells and thereby to increase the chances that subsequent immune mechanisms abort the nascent infection. This is also consistent with protection of macaques through systemic administration of inhibitors of reverse transcription or of entry (CCR5 ligand CMPD167), if treatment is started 24–48 hours after exposure to SIV or SHIV (HIV–SIV hybrids) and continued for 10–28 days [ 19, 20].

## Mucosal Immunity and Protection by Vaccines/Microbicides

What interventions can block viral transmission? As we have outlined, high viral load, specifically in genital fluids, raises the risk of HIV transmission. Thus, if vaccine-induced cell-mediated immunity (CMI) substantially reduced the viral load in genital fluids during primary infection, the vaccinee would not become a high-risk transmitter. Such a herd-immunity effect would reduce transmission by either gender. The majority of vaccines currently in clinical trials are aimed at eliciting CMI. However, CMI responses in the genital mucosa are largely unexplored because they are difficult to measure [ 4]. Intranasal immunization is the most efficient way to elicit genital tract immune responses in several animal models, although this approach potentially poses safety problems: Bell's palsy was observed following intranasal immunization with an influenza vaccine formulated with heat-labile Escherichia coli enterotoxin as an adjuvant [ 21, 22]. The lower genital tract is not a good inductive site, but immunogens delivered vaginally quickly reach the upper reproductive tract, providing an effective localized booster immunization [ 21, 22]. However, genital immunization could induce inflammation, which would enhance the risk of HIV infection. It is also noteworthy that systemic immunization on its own can induce mucosal immunity: papilloma virus infection of the anogenital mucosa can be prevented by an intramuscularly delivered vaccine [ 23]. The humoral component of that immunity is likely to be transudated IgG [ 22].

As complicating factors, the CD8 ^+^ cell numbers, cytokine, and possibly antibody secretion in the female genital tract vary with the menstrual cycle [ 4, 22]. Furthermore, vaccine protection needs to be effective in the presence of co-factors such as STDs, damaged mucosa (microabrasions), and vaginosis. STD treatment may enhance the effectiveness of vaccines and/or microbicides and should be further explored in clinical trials [ 4, 8].

Neutralizing antibodies have been shown to provide protection through passive immunization of animals [ 24], but their elicitation through active immunization has so far been the greatest hurdle in HIV vaccine development. Furthermore, the titers of neutralizing antibodies in vivo that are required to block vaginal transmission of HIV are many orders of magnitude greater than those measured in infected people, which in turn are much greater than any that have been induced by active immunization [ 24]. The problem of eliciting protective humoral immunity seems formidable. However, both mucosal and circulating antibodies to HIV may be beneficial through neutralization as well as other mechanisms [ 21].

HIV induces strong IgG responses in blood and in secreted body fluids (e.g., nasal, rectal, and vaginal secretions; semen, saliva, and tears). In contrast, IgA responses to HIV are low in these body fluids that typically contain high IgA levels. Importantly, human genital-tract secretions (semen and cervico-vaginal fluids) contain IgG derived largely from plasma. Thus, if systemic immunization resembles natural HIV infection, it would preferentially yield high levels of IgG in the genital tract. By combining mucosal (oral, rectal, and intranasal) with systemic immunization, one might achieve even higher levels of IgG in all compartments [ 21, 22]. To conclude, it may be feasible to induce strong humoral responses to HIV in the genital tract secretions, but how to achieve efficient neutralization remains unknown.

The lack of efficient vaccines that yield mucosal protection against HIV points to the potential benefits of microbicides and oral prophylaxis regimens, several of which are currently under development [ 8, 9]. First, a microbicide may be used as a substitute for mucosal neutralizing antibodies. This strategy could be combined with a vaccine that elicits efficient cellular immunity. Together they may render the population of infected founder cells too small for the infection to establish itself. Second, and more speculatively, HIV-1 immunogens, especially forms of the Env protein, added into microbicides, might improve mucosal immune responses: while they may not be effective by themselves, they may provide valuable mucosal boosts after systemic vaccination. To enhance immunogenicity, Toll-like receptor ligands might be added to the immunogen in the microbicide. A serious risk, however, is that this approach could yield inflammation and enhanced susceptibility to HIV-1 infection. Obviously, rigorous pre-clinical animal experimentation would be required to address such issues.

It is also possible that microbicides, particularly those that target the virus, when applied vaginally or rectally by infected individuals, would lower their infectiousness by reducing the infectivity of the virus exposed on their mucosae.

## Conclusion

Important questions about sexual transmission of HIV remain ( Box 1). Improved knowledge of host factors in both the transmitter and recipient that affect transmission will be crucial. It is essential to characterize HIV in genital secretions in terms of qualitative and quantitative differences between compartments and viral evolution during transmission. In conjunction with the exploration of increased microbicide efficiency, strategies for eliciting mucosal immunity will need to be pursued. In any case, both microbicides and vaccination would be compatible with condom use and risk-reducing behavior, which remain essential. Taken together, these measures, largely deriving from a biological understanding of the major mode of transmission, might have synergistic effects in curbing the pandemic.

**Five Landmark Papers on the Virus and Host during Sexual Transmission**

1. Wawer et al. [ 2]. This article demonstrates that the rate of HIV transmission is highest during early infection. Thus, interventions targeting early infection may have the greatest impact on curbing the epidemic.
2. Shattock and Moore [ 8]. This review discusses the concept of microbicides against the background of the receptors and cells involved in the mechanism of sexual transmission of HIV.
3. Miller et al. [ 17]. This study describes events in the mucosa and lymphatic tissue from hours to days after vaginal exposure of macaques to SIV, identifying a small window of opportunity for vaccines, microbicides, or other interventions to prevent or control infection.
4. Tsai et al. [ 20]. This paper shows that with the right timing and duration, systemic administration of ARV can prevent infection from becoming established.
5. Kozlowski and Neutra [ 21]. This review discusses the particular demands on mucosal immunity in attempts to prevent HIV transmission, and how such immunity may be elicited.

**Box 1. Future Directions for Research**

- Study transmission risk and viral load in plasma and genital secretions.
- Correlate cell-free and cell-associated viral load in genital secretions to risk of transmission.
- Investigate effect of vaccines on peak viral loads.
- Define the impact of co-pathogens (especially STDs) and hormones on vaccine and/or microbicide effectiveness in efficacy trials.
- Weigh the contribution of HIV–DC interaction to transmission in animal experiments.
- Isolate HIV from genital secretions.
- Identify mechanisms that restrict HIV replication in genital tract explants, including innate immunity.
- Evaluate mucosal boosting after systemic priming in order to induce genital immune responses.
- Evaluate inclusion of HIV-1 antigens, especially Env protein, as immunogens in microbicides.
- Improve mucosal immunogenicity—e.g., by incorporating Toll-like receptor ligands in vaccines/microbicides.

## Abbreviations

ARV: antiretroviral
CMI: cell-mediated immunity
DC: dendritic cell
IAVI: International AIDS Vaccine Initiative
SIV: simian immunodeficiency virus
STD: sexually transmitted disease
